# The publics of public health in Africa

**DOI:** 10.1080/09581596.2016.1254178

**Published:** 2016-12-01

**Authors:** Ann H. Kelly, Hayley MacGregor, Catherine M. Montgomery

**Affiliations:** ^a^ Faculty of Social Science and Public Policy, Department of Global Health & Social Medicine, School of Global Affairs, King’s College London, London; ^b^ Institute of Development Studies, University of Sussex, Brighton; ^c^ Health Experiences Research Group, Nuffield Department of Primary Care Health Sciences, University of Oxford, Oxford

How do we understand the *public* character of public health in contemporary Africa? What are the parameters of community engagement in health care delivery, medical research and disease control programmes? To what extent is public health in Africa a project led by African Governments? Through what political processes and deliberative practices can African publics influence the priorities of research in health sciences and interventions which aim in broad terms to improve the health of such publics? Drawing insight from empirical research conducted with African scientists, nurses, community members, clinical trialists and policy-makers, this special section examines the multiple ways in which *the public* comes into being around public health provisioning and investigation in sub-Saharan Africa, its role and political reach. Collectively, these papers show how contestation and negotiation around different ideas about *who the public is* and *what being public means* can lead to the emergence of conflicting understandings, with implications for who and what is seen to represent the public interest, and for the acceptance of research and other interventions.

The democratic potential of public participation has been a concern of development economics, moral philosophy, media studies and political science for quite some time. More recently, the task of enrolling the public in decision-making processes has become a salient issue for scientific and technological expertise: contemporary science policy emphasises the integration of public views into the assessment of research aims and outcomes (e.g. Jasanoff, 2010; Stilgoe, Lock, & Wilsdon, 2014). However, whose views should be included, how they are to be taken into account and to what end, are questions that perennially generate policy debate.

In recent years, social scientists have begun to investigate interactions between science and society beyond and between specific European and African locations (Craddock, Giles-Vernick, & Gunn, 2010; Montgomery, 2012). Seeking to ground political concepts such as ‘citizenship’, ‘public good’ and ‘representation’ in their diverse geographies and histories, scholars have pointed to the social solidarities that form around health issues and interventions (e.g. Kelly & Lezaun, 2014; Leach, Scoones, & Wynne, 2005; MacGregor, 2009). While on the one hand, these emergent publics seem to promise a new model for the relationship between government, science and citizen that cross cuts the local-global divide (Robins & Lieres, 2004), the tendency of foreign donors, global health professionals and activist organisations to regard emergent communities as the locus of democracy potentially undercuts national sovereignty and suggests unsettling parallels with both colonial rule and post-independence autocracy (e.g. Kamat, 2004; Lachenal, 2011; Nguyen, 2010). Furthermore, community-based organisations and NGOs, which proliferated in African settings in response to increases in global assistance for health, have marketed themselves as close to ‘local’ interests and giving voice to these, reinforced by donor constructions of the ‘community’ as an appropriate target for vertical interventions (Edstrom & MacGregor, 2010).

Some argue that the central role of NGOs in efforts to democratise African civil society points less to the empowerment of the grassroots than the triumph of a neoliberal model of governance (Dill, 2009; Kamat, 2004). For many African governments forced to restructure health activities in tune with the priorities of the World Bank, a *public* health system has only ever been a notional concept, or one greatly compromised by multiple social, material and political constraints. The effects of structural readjustment programmes on population health have received much academic attention (e.g. Pfeiffer & Chapman, 2010; Walt & Gilson, 1994); but equally important are the impacts of these reforms on the forms of credibility and accountability that ensure service quality. The provision of medical goods and services frequently occurs in the context of plural health markets and informal practices, prompting calls for partnerships between governments, citizen groups and the private sector to ensure some basic regulation (Peters & Bloom, 2012). As international research institutions, non-governmental organisations and aid-based charities perform the vital functions formally ascribed to the state, the *public* becomes both larger and smaller than the nation (Geissler, 2015). This problem of scale challenges how we imagine the legitimacy of public health practice and how we value its ends.

Renewed calls for commitment to universal health coverage have echoed within health systems research and beyond, such as in discussions linked to the Sustainable Development Goals. However, the challenges for many African governments of financing health ‘publics’ on the scale of the population remain substantial (Bennett, Osawa, & Rao, 2010). An argument has been made for incorporating approaches to ‘social accountability’ for monitoring of progress towards greater democratisation of a public health sector with respect to equality of access, quality of care and transparency in resource flows. Whilst the implementation of such mechanisms often remains at the level of technocratic instruments which fail to connect strategically to wider formal and informal political and institutional processes (Fox, 2014), the institution of measures such as health committees linked to the public health sector can at the very least point to a principle of state accountability to citizens (Cornwall & Shankland, 2008). The recent Ebola outbreak in West Africa has foregrounded questions about how to strengthen public health systems and drawn attention to notions of ‘resilience’, and the relationship between health system resilience and relations of trust between citizens and the state (Bloom, MacGregor, Mackenzie, & Sokpo, 2015; Wilkinson & Leach, 2014).

This special section engages with these debates about the political parameters of community collaboration in medical research, public engagement with health interventions and social accountability in health service delivery more generally. The central focus on ‘publics’ takes as its point of departure this concern with the shifting relationships between states, science, citizens and global and national public health. However, we aim to advance understanding by scrutinising these complex dynamics and the social, discursive and material links that come to be constituted so as to provoke thinking on the implications for research conduct and for public welfare. This collection addresses a gap in the literature by engaging critically with notions of the public with respect to health in African contexts and situating these understandings within ethnographic realities, historical imaginaries and policy perspectives. In so doing, it also addresses a need to integrate existing analytic perspectives with the practical implications of different framings of the public evinced in contemporary empirical research.

## Labour, legacies, infrastructures: defining the contours of public health’s publics in Africa

The papers in this special section describe well-intentioned attempts to enrich the social value of Global Health interventions: from empowering women to protect themselves from HIV (Montgomery & Pool, 2017) to building a community-based response to the Ebola outbreak (Folayan & Haire, 2017; Wilkinson & Fairhead, 2017) and from widening access to essential healthcare services (Koon, Smith, Ndetei, Mutiso, & Mendenhall, 2017) to creating platforms through which scientists can more easily access online data (Bezuidenhout, Kelly, Leonelli, & Rappert, 2017). Each paper highlights the gap – and in some cases the violent confrontation – between ideals of egalitarianism, empowerment, participation and communitarianism, and the lived realities of science and medicine on the ground. Collectively, the papers suggest the contours of a more thoughtful concept of publics than has previously been deployed in relation to public health in Africa. We wish to draw out three threads which are salient not only from a theoretical point of view, but also have immediate and practical import.

Firstly: rendering public the provision of health care services or research findings about health is a labour-intensive process. It is all too easy to conflate ‘public’ with ‘free’ and ‘open’, and in turn to assume this means ‘accessible’, when this is manifestly not the case (Hayden, 2010; Kelty, 2005). As Bezuidenhout and colleagues illustrate in their paper about Open Science, policy-makers’ attempts to close the digital divide by making more scientific research findings available online misses the point; it is not more material that is needed, but rather the material means of engaging with online content in the first place. Likewise, Koon et al. (2017) demonstrate the negative impact policies for universalising health care can have on staff morale when the additional labour it entails does not form part of the discourse or ensuing debate.

Secondly: publics do not take shape on *terra nulla* but in a specific time and place, whose political, economic and ecological histories saturate the present. The one-size-fits-all approach espoused by guidelines for community participation does a disservice to the diversity of participatory and informal accountability mechanisms which already exist. Likewise, clinical trials, being variable rather than case-based, have a tendency to empty places of their specificity by imagining them as interchangeable ‘sites’, with difference cast in terms of disease burden. When ‘the site’ becomes the starting point for community engagement, the legacies handed down from the past are rendered inconsequential. As Wilkinson and Fairhead’s analysis of resistance to Ebola interventions demonstrates, historical antecedents and relationships of trust and distrust are of great consequence. Rather than attribute these responses to culture, they instead show how patronage networks between central and local state actors account for differences in resistance to Ebola control measures. Their careful analysis of how the social orders of Mano River societies intersect with the political and administrative structures at a state level underscores the importance of historical context to the perceived legitimacy of present-day health interventions.

Thirdly: publics are not abstract and disembodied, but intertwined with the material infrastructures of participation. In their paper, Bezuidenhout and colleagues’ informants raise the issue of research funders’ unwillingness to fund computers. Their paper begs the question of how the ideals of Open Science should be realised in the absence of basic material infrastructure. Their paper echoes a long accepted dictum in Science and Technology Studies that objects have politics, and indeed, goes further to illustrate how scientific democracy is delimited by the unequal distribution of devices. Similarly alluding to what Marres and Lezaun (2011) call ‘material devices of the public’, Montgomery and Pool (2017) observe how both an experimental HIV prevention method, and the infrastructure surrounding its testing, differentially created participation in science along gendered lines. Both papers demonstrate that within the context of global public health, politics and democracy are not only ‘latent effects but also constituted forms’ (Marres, 2013, p. 423). As such, science funders and those who lead on collaborative projects need to acknowledge their role in creating spaces of participation and the associated politics of inclusion and exclusion.

Where such politics are ignored by public health workers, resistance is the inevitable outcome. This is true as much for the material politics of scientific practice as for the everyday politics of participation in society. Wilkinson and Fairhead’s (2017) paper illustrates this all too well, drawing out the continuities between the exploitation of a country’s natural resources and the extraction of scientific data from its people by outsiders. However, recognising the material and historical basis of social life is not to undercut its legitimacy: as Folayan and Haire (2017) remind us, communities have legitimate representatives who often speak up for the collective during times of crisis. Too often, they argue, bioethical discourse is tripped up by the thorny issue of representation; sometimes finding the right spokesperson is simply a matter of creating the space for one to step forward and acknowledging the local processes of accountability that legitimate who can speak for the collective.

## Beyond the abstract: addressing shifting realities and multiple publics

The discussion of publics often centres on absolutes and dichotomies, when the realities are partial, shifting and more nuanced. Furthermore, the tendency to speak in abstract terms often fails to acknowledge the situated nature of public engagement, both spatially and temporally. The situated nature of publics in relation to public health research should be seen as an opportunity, since change is most readily accomplished in specific places and within a particular moment in time. Thus, Bezuidenhout et al. (2017) propose a micro-funding scheme that would allow scientists to modify their research environments in ways that would facilitate greater engagement with digital resources. Montgomery and Pool (2017) suggest the need for trialists to work towards long-term forms of participation in science, which transcend the timelines of specific projects.

Kelty (2013) has highlighted the lack of conceptual clarity around the term ‘participation’; the same has been said of the varied collectivities and political subjectivities that constitute the public sphere (e.g. Bennett et al., 2010; Michael, 2002; Staeheli, 2008). Here, we do not propose yet another theoretical elaboration of the public or even offer to delimit its specific deliberative capacities within the context of Global Health. Rather, by offering an empirical re-description of the practices through which African publics come into being, our hope is to enrich discussion of participation in health sciences. The papers collected here prompt us to question how we can ensure the engagement of health researchers with institutions that have political legitimacy and that can help to appraise the potential harms and benefits of interventions which may have unintended consequences in contexts of inequality and rapid social change. Rather than singling out the meaning of publics, we are instead multiplying them. In so doing, we draw attention to the following practical implications: in projects, we need resourcing for the infrastructures of participation as well as acknowledgement of the labour involved in making things public. In public health services, institutions and mechanisms for greater social accountability should be cognisant of wider political landscapes and issues of inequality and representation. We need attention to the legacies of power and deliberative processes which shape people’s response to public health interventions and their willingness to trust in the services on offer. In research, we need careful descriptions of the ways in which health policies and programming structure, govern and enable participation.

## Funding

This work was supported by Economic and Social Research Council [grant number ES/L000350/1]; Wellcome Trust [grant number PHHPBD78]; Netherlands Organisation for Scientific Research [grant number 451-11-003]; ESRC STEPS Centre.

Ann H. Kelly *Faculty of Social Science and Public Policy, Department of Global Health & Social Medicine, School of Global Affairs, King’s College London, London* ann.kelly@kcl.ac.ukHayley MacGregor *Institute of Development Studies, University of Sussex, Brighton* Hayley.MacGregor@ids.ac.ukCatherine M. Montgomery *Health Experiences Research Group, Nuffield Department of Primary Care Health Sciences, University of Oxford, Oxford* catherine.montgomery@phc.ox.ac.uk
